# Long-Read High-Throughput Sequencing (HTS) Revealed That the Sf-Rhabdovirus X^+^ Genome Contains a 3.7 kb Internal Duplication

**DOI:** 10.3390/v15101998

**Published:** 2023-09-26

**Authors:** Hailun Ma, Trent J. Bosma, Arifa S. Khan

**Affiliations:** Division of Viral Products, Office of Vaccines Research and Review, Center for Biologics Evaluation and Research, U.S. Food and Drug Administration, Silver Spring, MD 20993, USA; hailun.ma@fda.hhs.gov (H.M.); trent.bosma@fda.hhs.gov (T.J.B.)

**Keywords:** *Spodoptera frugiperda*, Sf9 cell line, Sf-rhabdovirus X^+^, Sf-rhabdovirus X^+3.7^, Sf-rhabdovirus X^−^, high-throughput sequencing (HTS)

## Abstract

We previously reported a novel rhabdovirus produced from the *Spodoptera frugiperda* Sf9 cell line, designated as Sf-rhabdovirus X^+^ since it contained a unique accessory gene X. The Sf-rhabdovirus X^+^ genome sequence was generated using Sanger sequencing and short-read high-throughput sequencing (HTS). In this study, we have used long-read HTS technologies, PacBio’s single-molecule real-time sequencing and Oxford’s Nanopore RNA direct sequencing, to analyze the parent Sf9 cell line transcriptome and the virus RNA produced from an X^+^ cell clone, respectively. A unique 3.7 kb duplication was identified in the L gene between nucleotide position 8523 and 8524, preceded by a GA dinucleotide insertion. This duplication contained a partial G gene, the complete X gene, and a partial L gene, which extended from nucleotide positions 4767–8523 in the X^+^ virus. Thus, the X^+^ genome length is 17,361 nucleotides, and we have re-designated the virus as Sf-rhabdovirus X^+3.7^. The 3.7 kb duplication was found in all Sf9 cell clones producing the X^+^ variant virus. Furthermore, the Sf-rhabdovirus X^+3.7^ genome was stable at passage 30, which was the highest passage tested. These results highlight the importance of combining short-read and long-read technologies for accurately sequencing virus genomes using HTS.

## 1. Introduction

Next-generation or high-throughput sequencing (HTS) is a powerful technology for broad non-targeted microbial analysis. This has been applied for discovering new pathogen genomes in infectious diseases [1,2], understanding host–pathogen interactions [3,4,5], surveillance of epidemic virus variants [6,7], monitoring vaccine genetic consistency [8], and detecting viral adventitious agents in biological materials [9,10,11,12]. The first commercially available HTS technology was Roche-454 pyrosequencing, which was on the market in 2005 and has subsequently retired. Since then, several NGS platforms have been developed with differences in data volume, read length, error rate, and cost. These include short-read HTS technologies such as Illumina, SOLiD and Ion torrent, and the more recent, third-generation long-read sequencing technologies from Pacific BioSciences and Oxford Nanopore Technologies.

Our lab previously discovered a novel Sf-rhabdovirus in the Sf9 cell line from American Type Culture Collection (ATCC) using degenerate RT-PCR assays and Roche-454 short-read HTS technology [11]. The virus genome of the Sf-rhabdovirus X^+^ (GenBank accession number KF947078.1; designated as X^+^ in this paper) was constructed via de novo assembly of HTS reads obtained from total cell RNA prepared from Sf9 cell pellets at passage 20. The X^+^ genome sequence was verified using RT-PCR analysis and Sanger sequencing of overlapping fragments extending across the virus genome. Additionally, the viral genome sequence was confirmed by mapping Illumina HiSeq short-reads, which were obtained from total cellular RNA of a stored Sf9 cell pellet from the same lot that was used for 454 pyrosequencing [13]. Using the sequence of the Sf-rhabdovirus X^+^ genome, Maghodia et al. designed an RT-PCR assay, which amplified a virus genome in Sf21 cells with 99% nucleotide identity to Sf-rhabdovirus X^+^ [GenBank accession number MF536979.1; [14]]; however, the rhabdovirus in the Sf9 cells from their laboratory was an X gene variant with a 320 nt deletion, extending from the X gene into the intergenic region between X and L genes (GenBank accession number MF536978.1). The X^−^ variant virus was also reported in Sf9 cells by Schroeder et al. based on 454 pyrosequencing of Sf9 cells [GenBank accession number MH926031.1; [15]] and via RT-PCR analysis by Hashimoto et al. [16]. Further RT-PCR analysis in our laboratory confirmed the presence of the Sf-rhabdovirus X^−^ variant (designated as X^−^ in this paper) in ATCC Sf9 cells, which was the dominant variant at passage 39, whereas X^+^ was dominant at passage 20 [13].

In this study, we have identified and characterized a unique 3.7 kb duplication in the X^+^ variant virus using long-read HTS technologies. PacBio reads were obtained from total cell RNA prepared from the same lot of Sf9 cell pellets that were used in our earlier short-read sequencing analysis. Oxford Nanopore long-read sequencing reads were obtained from an X^+^ virus stock, which was prepared from cell-free supernatant of an X^+^ cell clone designated as Sf9-2-20 [13]. Consequently, we have re-designated the X^+^ virus as Sf-rhabdovirus X^+3.7^ (designated as X^+3.7^ in this paper), and report its genome length as 17,361 nucleotides. The stability of the 3.7 kb duplication was demonstrated with extended cell passage.

## 2. Materials and Methods

### 2.1. Sf9 Cells and Sf9 Cell Clones

The Sf9 cell line from ATCC, and all the cell clones derived from Sf9 cells have been described previously [11,13].

### 2.2. RT-PCR Analysis

All cellular RNAs used in this paper were used previously and stored at −80 °C. The quality and concentration of the cellular RNAs were determined using NanoDrop 1000 (Thermo Fisher Scientific, Waltham, MA, USA). For conventional RT-PCR, cDNA was synthesized from 1 µg total cellular RNA using iScript cDNA Synthesis Kit (Bio-rad, Hercules, CA, USA; catalogue no. 170-8890); PCR was performed using TaKaRa Ex Taq^TM^ kit (TaKaRa Bio USA, Inc., San Jose, CA, USA; catalogue no. RR001A) under the following conditions: 94 °C for 5 min, (94 °C for 30 s, 55 °C for 1 min, 72 °C for 1 min) × 35 cycles, followed by 72 °C for 10 min. For long-range RT-PCR, cDNA was synthesized using Maxima H minus First Strand cDNA synthesis Kit (Thermo Fisher Scientific, Waltham, MA, USA; catalogue no. K1651); PCR was performed using TaKaRa Ex Taq^TM^ kit under the following conditions: 94 °C for 5 min, (98 °C for 10 s and 68 °C for 10 min) × 35 cycles, followed by 72 °C for 10 min. PCR-amplified DNA fragments were gel-purified using Zymoclean gel DNA recovery kit (Zymo Research Corporation, Orange, CA, USA; catalogue no. D4001), and sequenced by Sanger sequencing or Illumina AmpliSeq at the CBER core facility. RT-PCR primers used in the study were as follows: Sf-rhabdo-F7, TCACATCTAGAGCTTGAAGACC (nt 6209–6230); Sf-rhabdo-R7, TCTGCTCTTGACCACCAGGA (nt 7323–7304); Sf-rhabdo-F9, CCATCTCCTTAGGTTTCCCAGA (nt 8157–8178); and Sf-rhabdo-R6, CACTGGCTGTGATGGTAGGT (nt 6332–6313). The locations of the primers are indicated based on the published X^+^ genome sequence (GenBank accession number KF947078).

### 2.3. PacBio HTS Sequencing 

Total cellular RNA was extracted at our lab from ATCC Sf9 cells at passage 20 using the RNeasy Plus Mini Kit (Qiagen, Gaithersburg, MD, USA; catalogue no. 74134) and sent to Institute for Genomic Sciences (University of Maryland, Baltimore, MD, USA) to perform PacBio sequencing. PacBio sequencing was carried out on the RSII (Pacific Biosciences, Menlo Park, CA, USA) after PacBio cDNA IsoSeq library preparation (polyA-enriched, size-selected). The SMRTbell template preparation kit was used to ligate hairpin adapters, and sequenced using PacBio’s P6-C4 chemistry using 240 min movies. The total raw reads obtained were 4,990,660 with an average length of 2869 bp. BLASTn analysis against Sf-rhabdovirus X^+^ (accession number KF947078.1) was performed using the CLC Genomics Workbench (version 9.0; Qiagen, Redwood City, CA, USA).

### 2.4. Oxford Nanopore HTS Sequencing

X^+3.7^/Sf9-2-20 filtered supernatant (10.4 mL) was ultracentrifuged (Beckman SWti40; Beckman Coulter, Indianapolis, IN, USA) at 125,000× *g* (27,000 rpm) at 4 °C for 2 h. Supernatant was removed leaving 500 µL which was divided into two samples (1 and 2) and total RNA was extracted using Trizol (Invitrogen, Waltham, MA, USA) following the manufacturer’s protocol, except for the addition of 5 µL of linear acrylamide (Invitrogen, Waltham, MA, USA) to aid the precipitation of the RNA. Each pellet was resuspended in 21 µL of nuclease-free water (Quality Biologicals, Gaithersburg, MD, USA). RNA was quantified using a Qubit RNA High Sensitivity Assay Kit with a Qubit R 2.0 Fluorometer (Thermo Fisher Scientific, Waltham, MA, USA), resulting in final concentrations of 132 ng/µL and 120 ng/µL for samples 1 and 2, respectively. Sample 2 was divided into two equal portions, and treated with AMPure XP beads (Beckman Coulter, Indianapolis, IN, USA) to remove short RNAs (<200 bp): 2A was treated with a ratio of 1.8 (AMPure XP beads to sample), whereas 2B was treated with a ratio of 0.6.

For Direct RNA Nanopore sequencing, poly(A) tails were added to the samples 1, 2A and 2B. RNA was incubated at 70 °C for 2 min and immediately placed on ice to avoid secondary structure formation. The Poly(A)-tailing reaction was performed in 50 µL reactions containing 5 µL 10× *E. coli* Poly(A) Polymerase Reaction Buffer, 8 µL ATP (10 mM) and 4 µL *E. coli* EPAP (NEB# M0276), and incubated at 37 °C for 10 min. Poly(A)-tailed RNA was then cleaned using 2.5 ratio of (by volume) of AMPure XP beads, and eluted with 21 µL nuclease free water for 2 min at room temperature. Cleaned Poly(A)-tailed RNA was incubated for 1 min at 70 °C and immediately placed on ice to remove secondary structure. For untreated sample 1, 1188 ng resulted in 1323 ng poly(A) RNA; for treated sample 2A, 1078 ng resulted in 1218 ng poly(A) RNA; for treated sample 2B, 206 ng resulted in 193 ng poly(A) RNA.

The cleaned Poly(A)-tailed RNA (9.5 µL) was used as input for library preparation and sequencing using Direct RNA Sequencing Protocol (SQK-RNA002 kit; Oxford Nanopore) for the MinION following the manufacturer’s instructions. Sequencing of the 3 libraries was performed on FLO-MIN106 flow cells on a MinION Mk1B for 8 h, 12 h and 22 h (samples 1, 2A and 2B), respectively. Sequences were generated using the guppy base caller version 6.2.1 with quality trimming at Q7. The supernatant RNA Nanopore raw read data of the Sf9-2-20 cell clone have been deposited at NCBI SRA under the accession number SRR25629548.

### 2.5. Illumina HiSeq HTS Sequencing 

Total cellular RNA from X^+3.7^/Sf9-2-20 cells and X^−^/Sf9-1A3 cells were extracted from cell pellets at passage 10 using RNeasy Plus Mini kit, and sent to the CBER core facility for whole transcriptome Illumina HiSeq sample processing and sequencing, which were described previously [13]. The total number of paired-end reads obtained were 350,862,670 for X^+3.7^/Sf9-2-20 and 339,525,936 for X^−^/Sf9-1A3, with an average length of 99 bp. Single nucleotide variant analysis was performed after mapping X^+3.7^/Sf9-2-20 or X^−^/Sf9-1A3 reads against the Sf-rhabdovirus X^+^ genome (accession number KF947078.1) using CLC Genomics Workbench (version 9.0; mapping parameters: length fraction = 0.5 and similarity fraction = 0.8). The whole transcriptome HiSeq raw read data of the Sf9-2-20 cell clone have been deposited at NCBI SRA under the accession number SRR25629549.

## 3. Results

Based on HTS long-read analysis, the previously reported Sf-rhabdovirus X^+^ genome was found to contain a 3.7 kb duplication, and therefore has a genome length of 17,361 nucleotides. Thus, we have re-designated this variant virus as X^+3.7^ and deposited its nucleotide sequence in NCBI GenBank (accession number OQ158798).

### 3.1. Identification of X^+3.7^ in Sf9 Cell Transcriptome

The genome of the original X^+^ variant (13,534 kb) was constructed using de novo assembly of 454 sequencing reads, which were obtained from ATCC Sf9 cellular RNA at passage 20 [11]. The sequence of the X^+^ genome was verified via RT-PCR and Sanger sequence analysis of overlapping fragments across the whole genome length and also confirmed by mapping the genome with Illumina HiSeq reads obtained from total cellular RNA prepared from the same lot of cell pellets used for the 454 sequencing.

**Figure 1:**
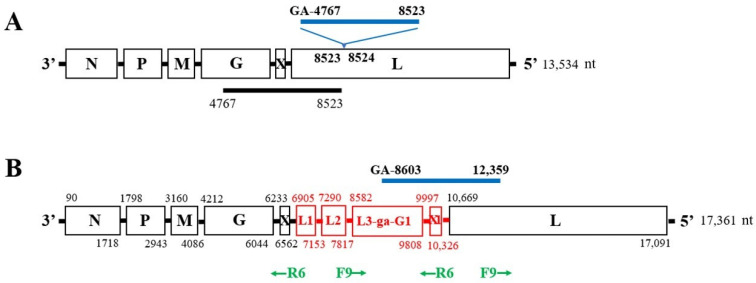
Genome of Sf-rhabdovirus X^+3.7^. (**A**) Location of the 3.7 kb duplication with respect to the Sf-rhabdovirus X^+^ genome (GenBank accession number KF947078). Primary sequences in the genome (nt 4767–8523) are indicated with a black bar. The 3.7 kb duplication, located at nt 8523, is shown by the blue bar along with the GA dinucleotide. (**B**) Genomic structure of the X^+3.7^ virus. The nucleotide positions indicated at the start and end of each ORF are based on the X^+3.7^ genome (GenBank accession number OQ158798). The four extra ORFs are shown in red between the X and L genes: L1, L2, L3-GA -G1 and X1. The regions between ORFs L1 and L2, L2 and L3-GA-G1, did not contain the putative conserved intergenic sequences. Location and orientation of primers F9 and R6 are shown in green arrows (F, forward; R, reverse). An RT-PCR product using the F9 and R6 primers can only be amplified from the X^+3.7^ genome.

We also analyzed PacBio sequence data, which were obtained using Sf9 cell RNA extracted from the same lot of stored Sf9 cell pellets that were used for the original virus assembly. BLASTn analysis of a total of 4,990,660 PacBio reads (average length 2869 bp) against the X^+^ virus genome (GenBank accession number KF947078) showed that 11,635 reads were aligned to X^+^ genome sequences (E-value 0.0). Detailed analysis of each PacBio read containing the X^+^ genome sequences indicated that 1292 reads covered the region extending from nt 2622 to 11,353 in the X^+^ genome and contained the 8523(L)-GA-4767(G) junction, which could be assembled to show the presence of 3.7 kb directly repeated sequences extending from nt 4767 in the G gene to nt 8523 in the L gene. Additionally, there was a dinucleotide GA insertion at 8523 (Figure 1A). The duplicated sequences resulted in four additional predicted ORFs along with the original G and L ORF (Figure 1B).

**Figure 2:**
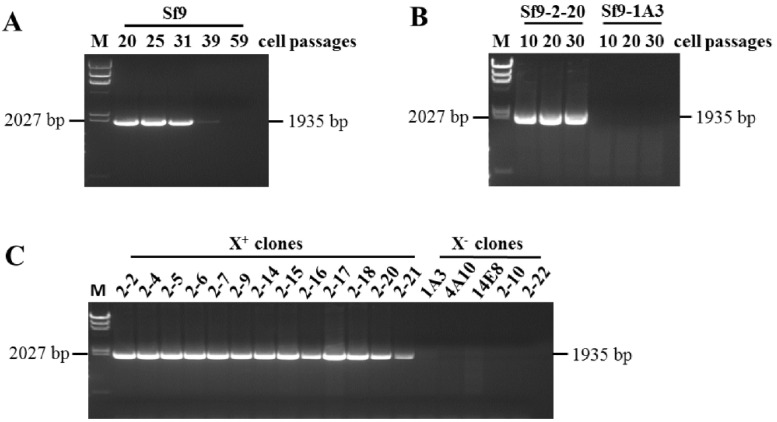
RT-PCR analysis of the 3.7 kb duplication using F9 and R6 primers. (**A**) Total RNAs from ATCC Sf9 cells were analyzed at the indicated cell passages: a 1935 bp fragment was amplified at passages 20, 25 and 31, with a dramatic decrease in the signal at passages 39 and 59. (**B**) Cellular RNAs were analyzed at passages 10, 20 and 30 from Sf9-13F12 cells infected with supernatant from the X^+^/clone Sf9-2-20, or with supernatant from the X^−^/clone Sf9-1A3: a 1935 bp fragment was amplified from the Sf-2-20 cells; no fragment was amplified from cells infected with the X^−^/Sf9-1A3 virus. (**C**) Cellular RNAs from X^+^ clones (2-2, 2-4, 2-5, 2-6, 2-7, 2-9, 2-14, 2-15, 2-16, 2-18, 2-20, 2-21) and X^−^ clones (1A3, 4A10, 14E8, 2-10, 2-22) were analyzed at passage 10: a 1935 bp fragment was amplified from the RNAs of all X^+^ cell clones; no fragment was amplified from the RNAs of the X^−^ cell clones. M: lambda DNA/HindIII maker with the 2027 bp fragment indicated on the left side of the panels.

To confirm the presence of the duplication, RT-PCR analysis was performed using the F9 primer combined with the R6 primer (Table 1 in ref. [13], and Figure 1B in this paper), which would only amplify sequences from the 3.7 kb duplication, producing a 1935 bp fragment. No RT-PCR product could be amplified with this primer combination in the absence of the 3.7 kb duplication. The Sf9 RNAs used for this analysis were the same as those used previously at passages 20, 25, 31, 39 and 59 [13]. Sanger sequencing of the RT-PCR amplified 1935 bp fragment (Figure 2A) showed that the duplication was present in Sf9 cellular RNA at passages 20 to 31 and decreased dramatically at passage 39. A similar pattern of expression was seen in the RT-PCR results of the X^+^ genome using X gene primers [13].

### 3.2. Identification of X^+3.7^ Produced by Sf9 X^+^ Cell Clones

We previously reported that the Sf9 cell line from ATCC is a heterogeneous population of X^+^ infected cells, X^−^ infected cells and Sf-rhabdovirus-negative cells. The X^+^ representative cell clone X^+^/Sf9-2-20, X^−^ representative cell clone X^−^/Sf9-1A3 and Sf-rhabdovirus negative representative cell clone Sf9-13F12 were isolated from ATCC Sf9 cells using limited dilution method [13]. To identify the presence of the duplication in the Sf9 cell clones, RT-PCR analysis was performed using F9 and R6 primers and the cellular RNA from Sf9-13F12 at passage 10, 20 and 30, following infection with the supernatant of X^+^/Sf9-2-20 or X^−^/Sf9-1A3 cells. A 1935 bp RT-PCR fragment was amplified from the X^+^/Sf9-2-20 infected cellular RNA, indicating the stable presence of the duplication (Figure 2B). Therefore, the X^+^/Sf9-2-20 cell clone is redesignated as X^+3.7^/Sf9-2-20. No RT-PCR fragment was amplified from the X^−^/Sf9-1A3 infected cellular RNA, indicating the absence of a virus containing the duplication. Furthermore, RT-PCR analysis was performed using the F9 and R6 primers for all X^+^ clones and selected X^−^ clones described previously. The 1935 bp fragment was amplified from the RNA of all X^+^ clones but was not present in the X^−^ clones (Figure 2C).

**Figure 3:**
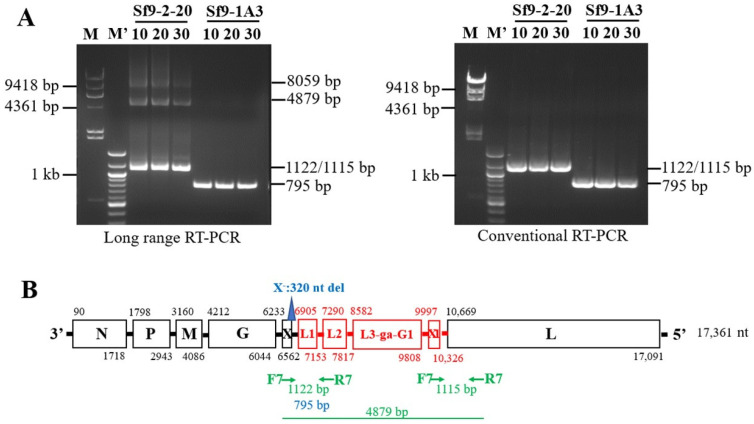
RT-PCR analysis of the 3.7 kb duplication using F7 and R7 primers. Total RNAs were analyzed at passage 10, 20 and 30 from Sf9-13F12 cells infected with supernatant from the X^+^/clone Sf9-2-20 or from the X^−^/clone Sf9-1A3. (**A**) Analysis using long-range PCR method (**left panel**) and conventional PCR method (**right panel**). Using conventional and long-range PCR methods, 1122/1115 bp fragments were amplified from primary and duplicated sequences, respectively, from cells infected with X^+3.7^/Sf9-2-20 and a 795 bp fragment was amplified from cell infected with X^−^/Sf9-1A3 (due to the 320 nt deletion indicated by the blue triangle in panel (**B**)). The expected 4879 bp fragment and an unexpected 8059 bp fragment were amplified from cells infected with X^+3.7^/Sf9-2-20 using long-range PCR method. Both fragments contained the duplication. Molecular size markers are shown: lane M, lambda DNA/HindIII maker with 9418 bp and 4361 bp fragments indicated; lane M’, 100-bp marker with 1000 bp (1 kb) fragment indicated. (**B**) Location of F7 and R7 primers in the X^+3.7^ virus genome and the size of the RT-PCR fragments are indicated. Green arrows, primers; blue triangle, 320 nt deletion present in the X^−^ virus variant.

RT-PCR assays were performed with F7 and R7 primers using previously described conventional PCR conditions [13]. Based on Sanger and high-throughput sequencing analysis, a doublet fragment (1122/1115 bp) was identified from the RNA of 13F12 cells infected with supernatant from X^+3.7^/Sf9-2-20 clone and a 795 bp fragment was amplified from the X^−^/Sf9-1A3 cell clone, due to the 320 nt deletion in the X gene (Figure 3A bottom panel and Figure 3B). To obtain longer PCR fragments which will include the duplicated sequences, long-range PCR conditions were used with the F7 and R7 primers. In addition to the 1122/1115 bp fragments, two larger fragments were amplified from the X^+3.7^/Sf-2-20 clone (Figure 3A, top panel). AmpliSeq sequencing showed that the expected 4879 bp fragment contained the 3.7 kb duplication and an unexpected 8059 bp fragment containing the duplication (located at nucleotide positions 6209–8523^4767–10,503 based on the reference Sf9 X^+^ genome, accession number KF947078; and at nucleotide positions 6281–14,339 based on the X^+3.7^ virus genome). Further analysis of the 1122/1115 bp fragment indicated that the 1122 bp fragment was amplified from the primary sequence, and the 1115 bp was amplified from the duplication sequences, which were distinct due to the 6 bp insertion at nt 7016 and G insertion at nt 7050 based on accession number KF947078 (described in Section 3.4).

### 3.3. Oxford Nanopore Sequencing of the X^+3.7^ Virus Produced from Sf-2-20 Cell Clone 

To get full length X^+3.7^ genome sequences, Oxford Nanopore direct RNA sequencing was performed on the total RNA extracted from cell-free supernatant of the X^+3.7^/Sf9-2-20 cell clone (NCBI SRA accession number SRR25629548). Different sample purification methods were tested to enrich the Sf-rhabdovirus sequences (see Section 2.4). The reads obtained from sample IDs 2B, 2A, and 1 from the three purification methods used are shown in Table 1. 

All of the raw reads were aligned against the reference rhabdovirus X^+^ genome using Minimap2 in CLC Genomics Workbench (v. 22.0.2) long-read plugin (Beta). The resulting hits were subjected to NCBI BLASTn analysis to identify the regions that contained insertions or duplications. Of the 883 total rhabdovirus reads that were identified, 9 had evidence of the duplicated region (Table 2), and none covered the region without the duplication. Reads 16,689 bp and 4634 bp were from sample 2A, reads 9135 bp, 11,631 bp, 4537 bp, 12,529 bp, and 11,247 bp were from sample 2B, and reads 2361 bp and 916 bp were from sample 1. Reads 16,689 bp and 12,529 bp (latter was reverse sequence) contained the entire duplication. The percent identity of the duplication sequence to the original sequence in the published X^+^ virus genome (accession number KF947078) is shown in Table 2. It should be noted that some of the differences in the sequence could be due to errors resulting from the long-read sequencing technology. The longest read was 16,689 bp, and it covered almost the full-length X^+3.7^ genome (nt 731–17,361 in X^+3.7^; accession number OQ158798). These results verified the results of the PacBio and RT-PCR analysis.

### 3.4. Characterization of X^+3.7^ Genome Structure

The 17 kb X^+3.7^ virus genome contained a 3757 nt duplication from nt 8603–12,359 that had 99% identity with the original (primary) sequences located at nt positions 4839–8600, which were 3762 nt in length (accession number OQ158798; Table 3). The duplication included the first 1691 nt of the L gene, the last 1206 nt of the G gene, and the entire X gene. Open reading frame (ORF) analysis found that X^+3.7^ generated four additional predicted ORFs between the X and L genes, which we designated as L1, L2, and L3-GA-G1 and X1 (Figure 1B, in red color). The location of the primary sequences and the GA dinucleotide insertion and duplication are indicated with a black bar and a blue bar, respectively, in Figure 1A. The location of putative genes in the reference Sf-rhabdovirus X^+^ virus genome (accession number KF947078) and in the X^+3.7^ virus genome (accession number OQ158798) is shown in Table 3. It was noted that putative conserved intergenic sequences that were present between the N, P, M, G, X, and L genes, were not found between L1 and L2, L2 and L3-GA-G1, thus indicating that it was unlikely that these putative ORFs could encode functional proteins.

Additionally, extra 72 nt were found at the 3′ end of the X^+3.7^ genome, which contained the putative conserved intergenic sequences GACACAAAAT/CC/AAT between 3′ and N. The additional 72 nt at the 3′ end of the virus genome with a 3 nt difference was also reported in the rhabdovirus genome by Schroeder et al. [15].

Twenty-three single nucleotide variants (SNVs) were found in the duplicated region by SNV analysis of the reads mapped using Illumina HiSeq data (NCBI SRA accession number SRR25629549) and by aligning Sanger sequences of RT-PCR fragments from both Sf9 and X^+3.7^/Sf9-2-20 cellular RNAs against the X^+^ virus genome (KF947078). Mapping was carried out using the CLC Genomics Workbench v9.0, specified to ignore the non-specific reads. To assign these mutations in the original or duplication region in the virus genome, PacBio reads and Nanopore reads, which comprised contiguous regions of both sequences, were manually reviewed to assign the position of the SNVs into their respective regions. A comparison of the SNVs in the primary and duplication in X^+3.7^ and the published X^+^ virus genome (KF947078) is shown in Table 4. The insertion of G at nt 7050 and the deletion of GA at nt 7699 generates the four unique ORFs predicted in X^+3.7^. The SNVs in the RT-PCR fragment from X^+3.7^/Sf-2-20 total cellular RNA using F9 and R6 primers, were confirmed by Sanger sequencing (Figure 2B). The conserved nucleotides in the G, X, and L genes in the X^+^ genome (KF947078) and the X^+3.7^ genome (OQ158798) are indicated in Table 4.

Additionally, comparison of the original X^+^ virus sequence obtained using 454-Roche (KF947078) with the X^+3.7^ genome sequence indicated 3 nucleotide differences in the latter located outside the primary and duplication region: G to A at position 2781, T to A at position 13,460 and A to G at position 17,196 (accession number OQ158798).

### 3.5. Analysis of HTS Long-Reads and Short-Reads for Assembly of Sf- Rhabdovirus X^+3.7^ Genome 

**Figure 4:**
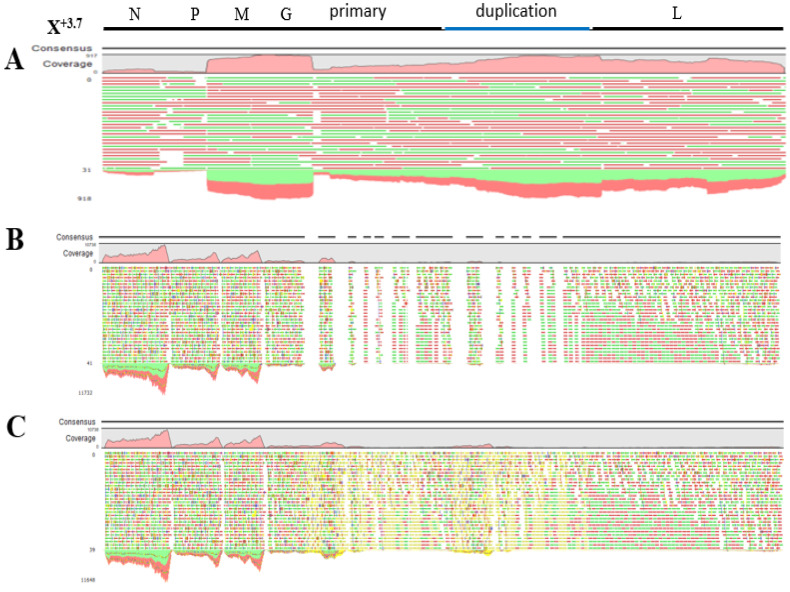
Mapping analysis of HTS long and short-reads using the Sf-rhabdovirus X^+3.7^ virus genome as reference sequence (accession number OQ158798). Reads were mapped against X^+3.7^ genome using CLC Genomics Workbench v9.0. The X^+3.7^ reference genome with N, P, M, G, and L genes and primary and duplication (blue) sequences are indicated at the top. (**A**) PacBio long-reads obtained from Sf9 cellular RNA at passage 20 were mapped, specifying to ignore the non-specific matches. Average length of mapped reads was 2369 bp and average depth coverage was 477 with a minimum and maximum coverage of 7 and 917, respectively. (**B**) Illumina Hiseq short-reads obtained from X^+3.7^/Sf9-2-20 cellular RNA were mapped, specifying to ignore the non-specific matches. Average length of mapped reads was 99 bp and average depth coverage was 1043 reads, ranging from 0 read and 10,738 reads for minimum and maximum coverage, respectively. (**C**) Same as (**B**), but mapped with random positioning of non-specific matches. The randomly positioned reads are indicated in yellow. The average depth coverage was 1189 reads, ranging from 0 read and 10,738 reads for minimum and maximum coverage, respectively. Reads mapping in the forward direction with respect to the X^+3.7^ genome used for the mapping are in green; reads mapping in the reverse direction are in red.

From a total of 4,990,660 reads, 3974 reads were mapped against the X^+3.7^ virus genome with PacBio long-reads, while ignoring non-specific matches and covering the entire genome length including the primary and duplication regions (consensus length of 17,361 bp) (Figure 4A). Long reads, containing both primary and duplication sequences, facilitated the characterization of the sequences and identified the duplication based upon nucleotide differences with the primary sequence in viral genome. In contrast, mapping with Illumina short-reads obtained from X^+3.7^/Sf9-2-20 cellular RNA (NCBI SRA accession number SRR25629548) using the ignored non-specific matches, covered 85% genome length (consensus sequence of 14,770 bp) with poor coverage of the primary and duplication regions (Figure 4B). From a total of 350,862,670 reads, 183,339 were mapped. The same data were also mapped, specifying that reads, which aligned in more than one position with equally good score be included and positioned randomly (Figure 4C). From a total of 350,862,670 reads, 208,969 were mapped. Full genome coverage was seen, and the consensus length was 17,348 bp, but the mapping was not confident since randomly positioning the Illumina reads inflated the results with a greater number of mapped reads and a higher coverage depth. 

## 4. Discussion

Rapid development of various NGS platforms have dramatically accelerated the discovery of new viruses. This has resulted in a continuing and substantial increase in the number of sequences and size of the VRL (viral) Division in GenBank. Different NGS platforms and bioinformatics strategies have been used for various applications in research, diagnostics, and product development. While the NGS short-read technologies have accomplished the task of reducing sequencing error rate, new bioinformatics tools continue to further improve the error rate of long-read sequencing. However, the assembly of short reads is still error-prone, particularly for repeat regions, and can even miss them in the assembly, even when a reference virus is used for mapping the reads [17]. This gap can be addressed using long-read NGS with a final correction of sequences carried out by mapping with the short reads. This hybrid assembly approach can successfully generate a confident genome sequence.

This study demonstrates the strength of using different NGS platforms and a hybrid assembly strategy for the bioinformatics, which resulted in the discovery of the 3.7 kb duplication in our previously published Sf9-rhabdovirus X^+^ genome [11]. The newly designated X^+3.7^ genome demonstrated the failure of using short reads for de novo assembly and mapping analysis for regions that contain repeated sequences with high identity. Previous de novo assembly of Illumina HiSeq short-reads from the Sf9-2-20 total cellular RNA did not identify the presence of the 3.7 kb duplication. Furthermore, in this study (Figure 4), mapping analysis against the X^+3.7^ genome sequences using short reads and specifying to ignore non-specific matches (i.e., reads aligning at more than one position with an equally good score) did not provide full coverage, particularly in the duplication region. However, reads containing the duplication junction (…8523^GA^4767…) were found with a manual review of the mapping data. Furthermore, re-analyzing the short-read data allowing for random mapping of the non-specific matches showed good coverage in the duplication region, but the results were not significant due to the random positioning of the reads. Interestingly, the long reads were mapped in the duplicated region even when the non-specific matches were ignored. Although the long-read platform was used to identify the 3.7 kb duplicated region, an accurate virus genome sequence could not be obtained due to the high error rate. Therefore, combining the short-read and long-read NGS platforms and bioinformatics analysis is a superior strategy for determining accurate viral genomes, particularly for capturing repeat regions.

Rhabdovirus genomes are generally 10.8–16.1 kb in size and contain five structural ORFs designated as N, P, M, G, L, and also some additional ORFs, whose functions are mostly unknown [18]. The largest rhabdovirus genome reported to date is Koolpinyah virus (16,133 nt), which was isolated from the blood of sentinel cattle from the genus *Ephemerovirus* [19]. Koolpinyah virus contained five additional ORFs (G_NS_-α1-α2-β-γ-δ) of about 4.4 kb between the G and L genes. The new genome of Sf-rhabdovirus X^+3.7^ is 17,361 nt and contains four additional predicted ORFs (L1-L2-L3/G1-X1) of about 3.7 kb between the X and L genes. Conserved signals, that were previously reported in the intergenic regions of the viral genome [11], were not found between the new ORFs in the X^+3.7^ genome. The functions of these additional ORFs are unknown. However, the presence of the large duplication could influence the downstream transcription from the L gene [20], leading to decreased replication of the Sf-rhabdovirus X^+3.7^. 

Gene duplication in the rhabdovirus genome has been reported, such as G and G_NS_ genes in bovine ephemeral fever rhabdovirus [21], and the U1, U2 and U3 genes in the Flanders hepavirus [22]. But these duplicated genes have very low amino acid identity. The 3.7 kb duplication sequence in the X^+3.7^ genome have 99% identity to the primary sequence. It is possible that recombination in the repeat region may be involved in generating the X^−^ variant.

## 5. Conclusions

A 3.7 kb duplication was discovered in the genome of the Sf-rhabdovirus X^+^ virus using long-read high-throughput sequencing technology. The presence of this large duplication in Sf-rhabdovirus X^+3.7^ could not be detected using Roche-454, Illumina HTS, and RT-PCR assays. The inability to identify this large duplication in previous studies highlights the difficulty of assembling viral genomes that contain repeat regions from short-read sequencing data, and demonstrates the utility of combining both long-read and short-read HTS data to obtain complete and accurate viral genome structure and sequences.

## Figures and Tables

**Table 1:** Nanopore long-reads from each of the purification method.

Sample IDs	Total No. of Reads	No. of Reads Past Q7 Filter	Average Q7 Past Read Length (bp)	No. of Rhabdovirus Reads (CLC)	Average Rhabdovirus Read Length (bp)
2B	28,062	5679	831	562	2481
2A	233,281	93,287	570	92	2744
1	571,301	193,530	509	229	2025
Combined		292,496		883	

**Table 2:** Nanopore long-reads containing the duplication sequences in X^+3.7^ in Sf9-2-20 cell supernatant RNA.

Read Length (bp)	Aligned Region in KF947078 3→5′	Identity (%) ^1^	Gaps (%) ^1^
16,689	659–8523^GA^4767–13,526 (338) ^2^	92.1	5.5
12,529-R ^3^	(83)-8523^GA^4767–8673	93.0	4.9
11,631	353–8523^GA^4767–8295	93.5	4.6
11,247-R	(84)-8523^GA^4767–7555	91.9	5.6
9135	(65)-8523^GA^4767–5650	88.2	8.0
4634-R	4407–8523^GA^4767–5375	91.1	6.5
4537	7054–8523^GA^4767–7613	91.4	6.2
2361	6297–8523^GA^4767–4895	88.6	7.2
916-R	8096–8523^GA^4767–5275	92.8	5.7

^1^ Identity and gap from BLASTn results against the X^+^ virus sequences, accession number KF947078. ^2^ number in ( ) indicates the additional sequences at 3′ end and 5′ ends found in this study compared to the previously published X^+^ virus genome sequence (KF947078). ^3^ R, reverse RNA strand.

**Table 3:** Locations of putative genes in the Sf-rhabdovirus X^+^ virus genome (accession number KF947078) and X^+3.7^ virus genome (accession number OQ158798).

Putative Genes	Location in KF947078 (nt)	Location in X^+3.7^ (nt)
N	81–1646	90–1718
P	1726–2871	1798–2943
M	3088–4014	3160–4086
G	4140–5972	4212–6044
X	6161–6490	6233–6562
**L1**	**6833–7074**	**6905–7153**
**L2**	**7211–7740**	**7290–7817**
**L3/G1**	**8505–8523^GA^4767–5972**	**8582–9808**
**X1**	**6161–6490**	**9997–10,326**
L	6833–13,255	10,669–17,091

New ORFs are indicated in bold.

**Table 4:** Comparison of nucleotide changes in the duplication sequences of X^+3.7^ and the published Sf-rhabdovirus X^+^.

Reference X^+^ Genome		X^+3.7^ Genome			
		Primary Sequence		Duplication Sequence	
Location (ORF)	nt	Location (ORF)	nt	Location (ORF)	nt
4878 (G)	T	4950 (G)	T	**8714 (L3/G1)**	**C**
4905 (G)	C	4977 (G)	C	**8741 (L3/G1)**	**T**
4908 (G)	G	4980 (G)	G	**8744 (L3/G1)**	**A**
5003 (G)	G	5075 (G)	G	**8839 (L3/G1)**	**A**
5036 (G)	G	5108 (G)	G	**8872 (L3/G1)**	**A**
5578 (G)	G	5650 (G)	G	**9414 (L3/G1)**	**A**
5687 (G)	G	5759 (G)	G	**9523 (L3/G1)**	**A**
5817 (G)	A	5889 (G)	A	**9653 (L3/G1)**	**G**
6333 (X)	C	6405 (X)	C	**10,169 (X1)**	**A**
6354 (X)	A	6426 (X)	A	**10,190 (X1)**	**T**
6732 (X-L)	C	**6804 (X-L)**	**T**	10,568 (X1-L)	C
7016^7017 (L)	-	**7089–7094 (L1)**	**AAAACC**	10,852^10,853 (L)	-
7018 (L)	A	**7096 (L1)**	**C**	10,854 (L)	A
7050^7051 (L)	-	**7129 (L1)**	**G**	10,886^10,887 (L)	-
7452 (L)	C	**7531 (L2)**	**T**	11,288 (L)	C
7624 (L)	T	**7703 (L2)**	**G**	11,460 (L)	T
7699–7700 (L)	AG	**7777^7778 (L2)**	**--**	11,535–11,536 (L)	AG
8052 (L)	C	**8129 (L2-L3/G1)**	**T**	11,888 (L)	C
8070 (L)	G	**8147 (L2-L3/G1)**	**A**	11,906 (L)	G
8104 (L)	G	**8181 (L2-L3/G1)**	**T**	11,940 (L)	G
8164 (L)	C	**8241 (L2-L3/G1)**	**T**	12,000 (L)	C
8189 (L)	G	**8266 (L2-L3/G1)**	**A**	12,025 (L)	G
8351 (L)	G	**8428 (L2-L3/G1)**	**A**	12,187 (L)	G

Nucleotide differences in the X^+^ reference virus genome (KF947078) and in the primary sequence and duplication in X^+3.7^ virus genome (OQ158798) are indicated in bold.

## Data Availability

The Sf-rhabdovirus X^+3.7^ genome has been deposited at NCBI Nucleotide under the accession number OQ158798. The whole transcriptome HTS raw read data of the Sf9-2-20 cell clone using the Illumina HiSeq technology have been deposited at NCBI SRA under the accession number SRR25629549. The supernatant RNA HTS raw read data of the Sf9-2-20 cell clone using the Nanopore technology has been deposited at NCBI SRA under the accession number SRR25629548. Other HTS raw data is available upon request.
